# Supplementary material for: MetaRibo-Seq measures translation in microbiomes
Source: Nat Commun. 2020 Jun 29;11:3268. doi: 10.1038/s41467-020-17081-z (PMC7324362; doi:10.1038/s41467-020-17081-z)
Supplement: Supplementary file 10 — Supplementary Data 7 [file 41467_2020_17081_MOESM10_ESM.zip › File2/Confidence_High_Taxonomy/174242_out.krona.html]

Javascript must be enabled to view this page.

members
magnitude
magnitudeUnassigned
count
unassigned
taxon
rank

174242\_out

2

2
superkingdom
2

2
1239
phylum

2
909932
class

1843489
order
2

family
31977
2

1

SRS893348\_contig\_number\_contig-100\_1133.100200
29465
2
genus

39777
species

SRS144560\_contig\_number\_23395
1
